# Non-invasive vessel fractional flow reserve versus fractional flow reserve guidance to revascularize intermediate coronary stenosis (LIPSIA-STRATEGY) trial: study protocol for a randomized controlled trial

**DOI:** 10.1186/s13063-026-09652-0

**Published:** 2026-04-01

**Authors:** Nicolas Majunke, Steffen Desch, Tobias Kister, Maria Buske, Janine Pöss, Hans-Josef Feistritzer, Sandra Erbs, Nadine Hösler, Janine Wolff, Steffen Schneider, Taoufik Ouarrak, Felix Woitek, Karsten Lenk, Holger Nef, Oliver Dörr, Samuel Sossalla, Stephan Achenbach, Mohamed Marwan, Dimitrios Barlagiannis, Michael Haude, Amir Abbas Mahabadi, Tienush Rassaf, Holger Thiele

**Affiliations:** 1 Department of Internal Medicine/Cardiology, Heart Center Leipzig at University of Leipzig, Leipzig, Germany; 2 Leipzig Heart Science, Leipzig, Germany; 3 Institut für Herzinfarktforschung, Ludwigshafen am Rhein, Germany; 4 Heart Center Dresden – Technische Universität Dresden, Dresden, Germany; 5 University Clinic Leipzig, Klinik und Poliklinik für Kardiologie, Leipzig, Germany; 6 Cardiology Department, Heart Center, Segeberger Kliniken GmbH, Bad Segeberg, Germany; 7 CCB Am Markuskrankenhaus, Med. Klinik III, Agaplesion Markuskrankenhaus, Frankfurt Am Main, Germany; 8 Department of Internal Medicine/Cardiology, University Clinic Giessen, Giessen, Germany; 9 Department of Cardiology, Friedrich-Alexander-Universität Erlangen-Nürnberg (FAU), Erlangen-Nürnberg, Germany; 10 Medical Clinic I, Rheinland Klinikum Neuss GmbH, Lukaskrankenhaus, Neuss, Germany; 11 Department of Cardiology and Vascular Medicine, West German Heart and Vascular Center, University Hospital Essen, Essen, Germany

**Keywords:** Angiographically intermediate coronary stenoses, Stable angina, Acute coronary syndrome, Fractional flow reserve

## Abstract

**Background:**

Current guidelines on coronary revascularization support the use of wire-based coronary physiology measurements to guide decision making in patients with coronary artery stenoses. Nevertheless, the use of these techniques in clinical practice is variable and its application worldwide remains limited by its requirement for the use of an intracoronary pressure wire and prolonged procedure time. Recently, angiography-based wire-free techniques to estimate fractional flow reserve (FFR) values have been introduced. These developments may translate towards more physiology-guided intervention bearing the potential to improve clinical outcomes in patients with stable coronary artery disease (CAD).

**Methods:**

The LIPSIA-STRATEGY trial is a randomized controlled, investigator-initiated, multicenter, open-label study. A total of 1054 eligible patients will be randomized 1:1 to coronary revascularization based on angiography-derived vessel fractional flow reserve (vFFR) or revascularization based on FFR obtained by pressure wire measurements. The major inclusion criterion is the presence of visually assessed intermediate coronary artery stenoses in one or more native major epicardial coronary arteries in the setting of stable angina or an acute coronary syndrome (ACS). In patients with an ACS, only non-culprit vessels will be considered for inclusion. The primary endpoint is the occurrence of major adverse cardiovascular events (MACE) during the first year after randomization.

**Discussion:**

The LIPSIA-STRATEGY trial will be the first to compare angiography-derived vFFR with invasive FFR with respect to clinical outcomes in patients with intermediate coronary lesions.

**Trial registration:**

ClinicalTrials.gov NCT03497637. Registered 2018–04-13.

## Introduction

### Background and rationale {6a}

Visual assessment of invasive, selective coronary angiograms alone is insufficient to determine the hemodynamic significance of intermediate coronary stenoses [1]. Based on a robust body of evidence, current guidelines recommend pressure-wire-guided revascularization in patients with intermediate coronary stenoses and absent non-invasive evidence of ischemia [2–4].

To date, several wire-based techniques to determine invasive coronary physiology are available which can be broadly classified as follows: (1) Classical measurement of the fractional flow reserve (FFR) after administration of a hyperemic agent; (2) Indices which do not require hyperemia, such as the instantaneous wave-free ratio (iFR), the diastolic pressure ratio (dPR), the resting full-cycle ratio (RFR), and the diastolic hyperemia-free ratio (DFR). While measurement of non-hyperemic pressure ratios is easier and faster to perform than classical FFR, they still rely on placement of an intracoronary pressure-wire. Possibly due to the increased time and effort required, the uptake of pressure-wire-guided revascularization in clinical routine has been limited [5].

To further simplify the hemodynamic evaluation of intermediate coronary stenosis without the need for wire placement, angiography-based wire-free techniques to estimate FFR have been introduced. Briefly, dedicated software reconstructs a 3D model of the vessel and the lesion. Based on this reconstruction and the invasively measured aortic root pressure, angiography-based FFR is calculated. The nomenclature for angiography-derived FFR measurements varies according to the software utilized, such as Medis, The Netherlands (Quantitative Flow Ratio, QFR), CathWorks, Kfar-Saba, Israel (FFR-angio), Rainmed Ltd, Suzhou, China (FlashAngio, caFFR) or Pie Medical Imaging, Maastricht, The Netherlands (vessel FFR, vFFR). Observational studies showed good diagnostic accuracy of different angiography-derived FFR with sensitivities ranging from 78 to 97% and specificities ranging from 74 to 98% in comparison with pressure-wire measured FFR as a reference [6–13].

These new techniques could lead to an increased rate of physiology-guided intervention bearing the potential to improve clinical outcomes in patients with coronary artery disease (CAD). It is of major importance that the clinical outcome following revascularization based on angiography-derived FFR is noninferior to the outcome following wire-based FFR measurements in order to provide a sufficient basis for the application of non-invasive FFR algorithms in clinical practice.

Therefore, the LIPSIA-STRATEGY trial will compare an image-based vFFR methodology with standard pressure-wire derived FFR in terms of clinical outcomes.

### Objectives {7}

The primary objective of the LIPSIA-STRATEGY trial is to determine whether non-invasive angiography-derived vFFR is non-inferior to conventional invasive wire-guided FFR regarding the clinical outcome of patients undergoing physiology-guided percutaneous coronary intervention (PCI) of intermediate coronary lesions.

### Trial design {8}

**Fig. 1:**
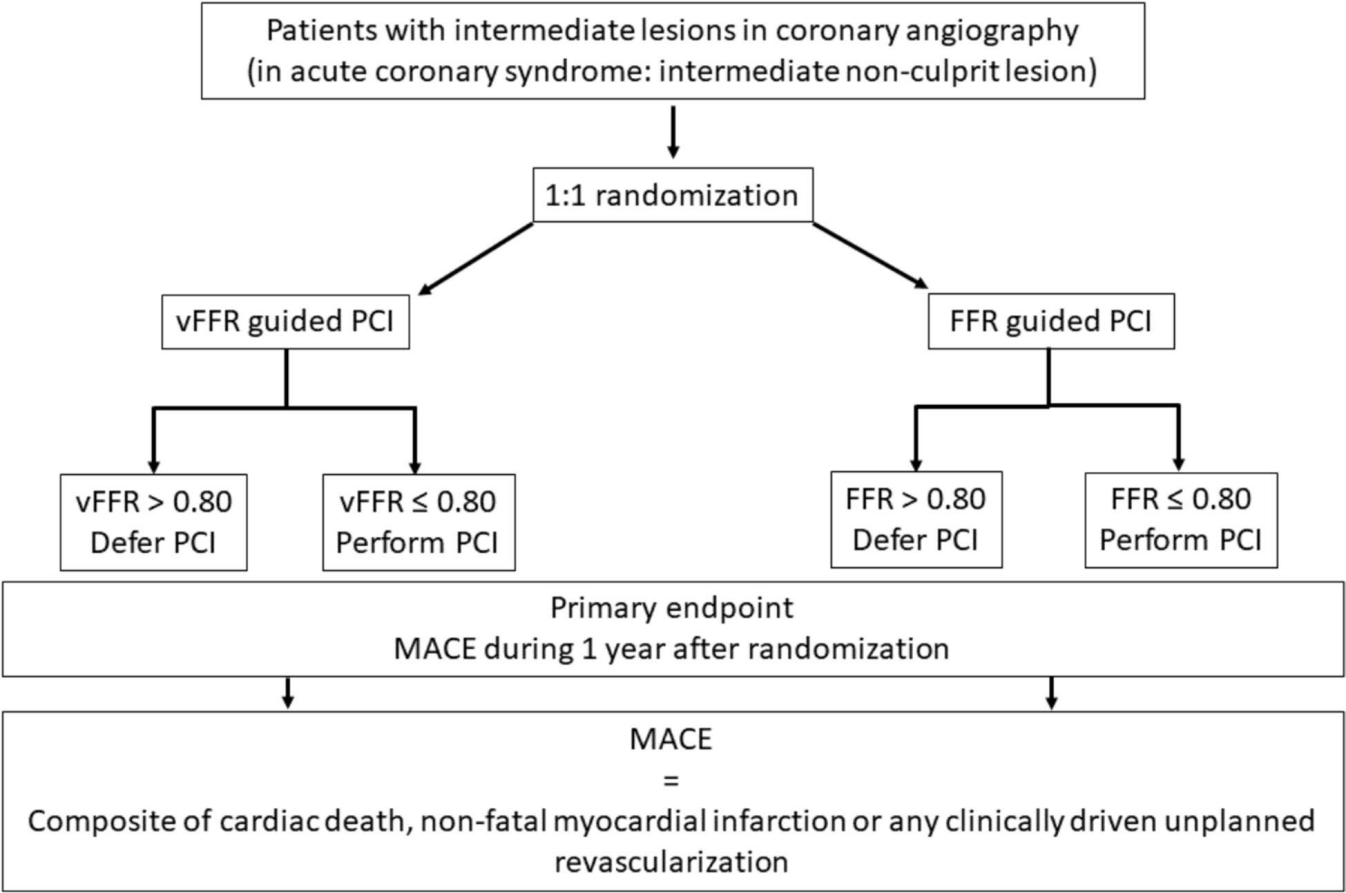
Study flow chart. PCI = percutaneous coronary intervention. FFR = Fractional Flow Reserve. vFFR = vessel Fractional Flow Reserve. CABG = Coronary Artery Bypass Graft. MACE = Major Adverse Cardiac Event

The LIPSIA-STRATEGY trial is a randomized controlled, investigator-initiated, multicenter, open-label study conducted at 8 tertiary hospitals in Germany. The trial flow chart is shown in Fig. 1. Patients will be randomized to revascularization decisions based either on vFFR calculated from angiographic images or on conventional wire-based FFR measurements.

## Methods: participants, interventions, and outcomes

### Study setting {9}

The LIPSIA-STRATEGY will be performed at 8 tertiary hospitals in Germany. The participating centers are high-volume tertiary care centers with extensive expertise in treatment of patients with CAD and are extensively experienced in performing clinical studies.

### Eligibility criteria {10}

#### Inclusion criteria

- Age > 18 years
- Willing to participate and able to understand, read, and sign the informed consent document before the planned procedure
- Eligible for PCI following invasive coronary angiography
- CAD in one or more native major epicardial vessels or their branches by coronary angiogram with visually assessed de novo coronary stenosis for which the investigator sees the need for a physiology-based revascularization decision (typically 40–090% diameter stenosis)
- Stable angina pectoris ACS (non-culprit vessels only and outside of primary intervention during acute STEMI or non-ST-elevation acute coronary syndrome [NSTE-ACS])

#### Exclusion criteria

- Previous coronary artery bypass graft surgery (CABG) with patent grafts to the interrogated vessel
- Tandem coronary artery stenosis separated by more than 10 mm which require separate pressure guidewire interrogation or PCI (not to be assessed or treated as a single stenosis)
- Total coronary occlusions
- Hemodynamic instability (Killip class III–IV)
- Heavily calcified or tortuous vessels
- Terminal disease with life expectancy of less than 12 months
- STEMI within 24 h of primary procedure
- Severe valvular heart disease
- ACS patients with difficulty in determining the culprit lesion
- Significant contraindication to adenosine administration (e.g., asthma)
- Pregnancy
- Participation in another interventional study
- Inadequate visualization of the target vessel (e.g., severe vessel overlap or tortuosity) which does not allow proper measurement of angiography-based FFR

### Who will take informed consent? {26a}

All eligible patients will be informed prior diagnostic coronary angiography through one of the investigating physicians. Investigators will inform the patient orally and in writing about the scope and purpose, rights, duties, and possible risks and benefits of the study in lay language. It will be explicitly highlighted that non-participation in the trial or a potential withdrawal of a prior given consent can be carried out at all times with no subsequent disadvantages and that management of all data will be performed according to data privacy policy. Patients unable to give written informed consent due to unconsciousness cannot be included in the study. Informed consent will be obtained according to the GCP-guidelines and the Declaration of Helsinki. Acceptance to participate in the study is obtained by signing a specific consent form and countersignature of the informing physician. After diagnostic coronary angiography patients who do not meet the exclusion criteria but fulfil the inclusion criteria will be orally informed about now participating in the study and will be randomized to either vFFR or FFR-guided therapy. If diagnostic coronary angiography disqualifies patients from inclusion in the study, patients will be orally informed about this and patient’s participation in the study will be discontinued. The procedure will be continued according to the decision of the individual investigator.

## Interventions

### Explanation for the choice of comparators {6b}

Invasive measurement of the FFR is the reference method for functional assessment of lesion severity in intermediate-grade stenosis (typically around 40–90% stenosis) without evidence of ischemia in non-invasive testing [2]. However, invasive FFR measurement incurs extra costs (FFR equipment and drugs used for hyperemia), produces side effects and is time-consuming. For these reasons and others, FFR utilization has remained low [5]. There is a need for alternative solutions of rapid physiologic assessment of coronary stenoses without need for invasive pressure wires. Different systems have been developed to predict FFR based on coronary angiographic images, eliminating pressure wires or hyperemic agents [6–13]. Vessel-FFR (vFFR, Pie Medical, Maastricht, The Netherlands) is a novel method for evaluating the functional significance of coronary stenosis by calculation of the pressure drop in the vessel based on computation of two angiographic projections. The ability to derive FFR values from routinely performed coronary angiograms, without the practical drawbacks that limit invasive techniques, could have an important impact on daily clinical practice [7, 12].

### Intervention description {11a}

#### Pressure-wire-based FFR measurement

If a patient is randomized to the FFR-group, a standard pressure-derived FFR measurement will be performed to assess the hemodynamic significance of the coronary stenosis [14].

Briefly, after the pressure sensor of the wire is zeroed and equalized to aortic pressure, a guide catheter without side holes is used to engage the coronary artery. After pressure equalization, the pressure wire is positioned distal to the stenosis with the sensor in the distal third of the target artery. Thereafter, adenosine is administered to induce stable hyperemia (intracoronary bolus of 200 μg for the s and 100 μg for the right coronary artery or intravenous route of 140 μg/kg per minute at the discretion of the investigator). Once steady-state hyperemia is achieved, FFR is calculated as the ratio of mean distal coronary artery pressure to mean aortic pressure across the complete cardiac cycle.

#### Angiography-based vFFR measurement

Computational fluid dynamics (CFD) is the most common mathematical solution for the investigation of blood flow patterns in coronary arteries. CFD solves numerically the fundamental Navier–Stokes equations that describe the flow of viscous fluids and is therefore able to analyze blood flow velocity and pressure between any points in the coronary artery [15]. CFD coupled with a 3-dimensional anatomic model of the coronary angiogram enables measurement of non-invasive FFR [16, 17].

Since this approach can be time consuming and requires long computation times, it is inappropriate in providing immediate measurements for fast clinical decision making. Therefore, faster and simpler commercial methods relying on the laws of Bernoulli and/or Poiseuille for instantaneous calculation of angiography-based FFR were introduced [6–13].

The vFFR methodology (CAAS, Pie Medical Imaging, Maastricht, the Netherlands) used in this study uses simplified analytical equations based on Bernoulli and Poiseuille to calculate angiography-based vFFR. The software requires two angiograms at least 30° apart in rotation or angulation to obtain a 3D model of the diseased coronary artery and the patient’s invasively measured aortic root pressure which is used as an inlet boundary condition. The algorithm within the software incorporates the 3D model and patient’s specific aortic root pressure to calculate the pressure drop within the diseased coronary artery [7].

In practice, if a patient is randomized to the vFFR-group, computation of vFFR will be performed immediately on-site by an experienced and trained operator. Therefore, two angiographic projections of the target vessel with at least 30° difference in rotation and / or angulation will be obtained and directly exported to the CAAS workstation. After reconstruction of the diseased vessel in 3D based on the two angiographic projections vFFR is automatically calculated incorporating the invasively measured aortic root pressure.

**Fig. 2:**
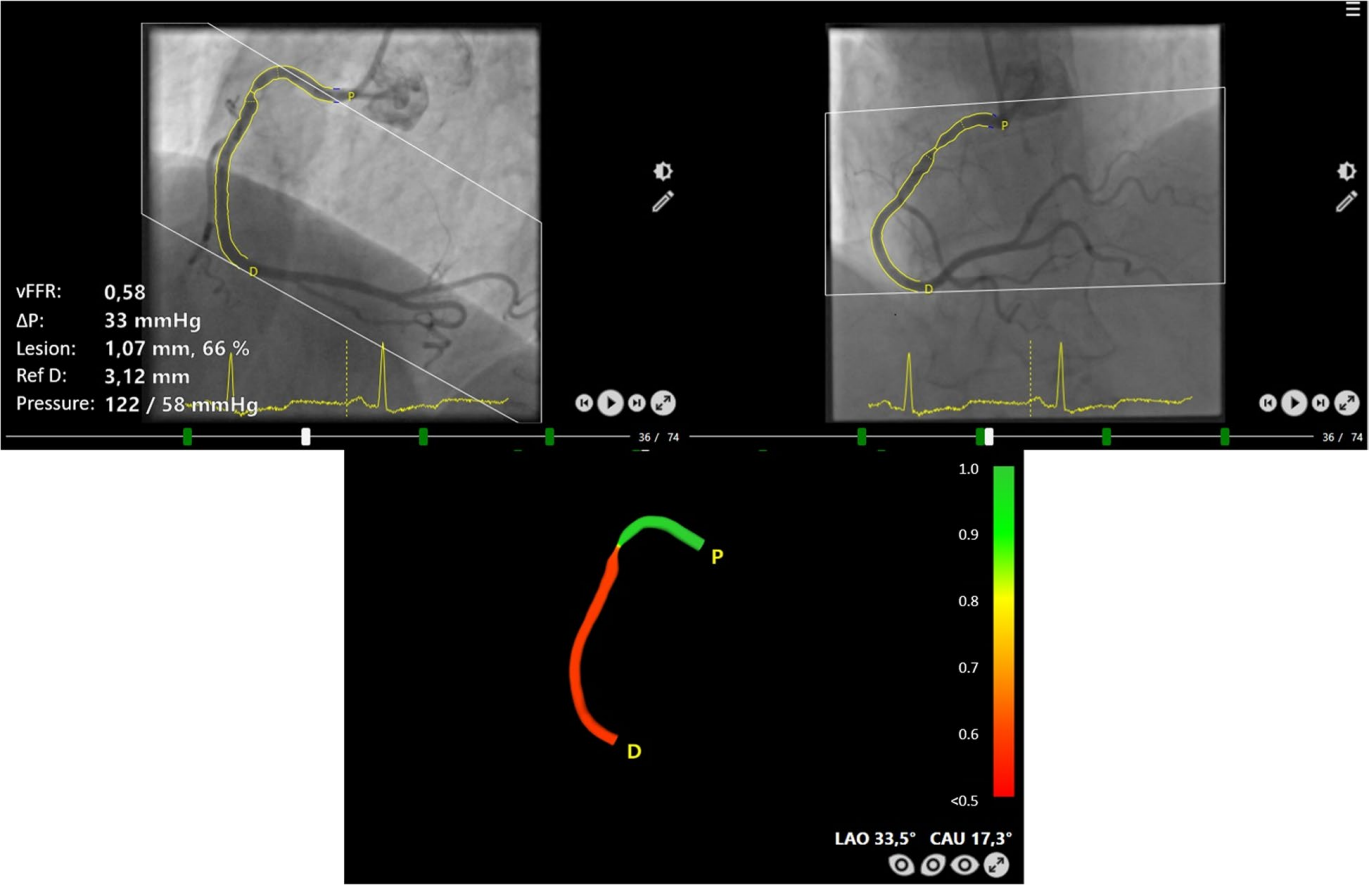
Example for computation of vFFR of a stenosis of the right coronary artery using 3D coronary reconstruction and invasively measured aortic root pressure, resulting in a vFFR value of 0.58 indicating hemodynamic significance

The vFFR values at each point along the vessel will be color-coded and superimposed on the 3D epicardial model (Fig. 2).

Prespecified thresholds to proceed with coronary revascularization are defined as vFFR or FFR ≤ 0.80. For values of 0.81 or more, a deferred strategy of optimal medical therapy will be applied. Any cross-over of patients will be considered as a protocol violation. The use of either vFFR or FFR will be the only difference between the two treatment arms. Antiplatelet therapy and additional medical treatment of CAD will be administered according to current guidelines.

### Criteria for discontinuing or modifying allocated interventions {11b}

For the individual patient:

The individual patient can prematurely terminate the study at any time. In general, this should be avoided. Premature termination of the study is only possible by withdrawal of the patient’s consent. Violation of the protocol does not lead to termination of the study for the individual patient. In case of premature termination, the reasons and circumstances, as well as the clinical state of the patient should be documented. This is especially important in the setting of a serious adverse event (SAE) leading to withdrawal of consent. In case patients are ineligible for further analysis during the follow-up period (= no contact can be established neither personally to the patient nor through family members) the exact time of the loss of contact should be documented (if possible with an explanatory statement, e.g., relocation). According to the intention to treat analysis, all events and parameters will be evaluated until the last date of contact.

For the entire study:

The final decision on a premature discontinuation of the study lies within the discretion of the coordinating investigator. The evaluation of events will be carried out by the steering committee in regular intervals.

The study can be prematurely discontinued by the coordinating investigator in case of:

- Unacceptable high rate of SAE based on the judgment of the steering committee by reviewing the individual cases.
- Unexpected adverse events leading to revision of the risk–benefit evaluation based on the judgment of the steering committee by reviewing the individual cases.
- Insufficient recruitment rate.

At an investigating center:

The study can be discontinued at an investigating center in case of:

- Failure to comply with the study protocol.
- Insufficient data quality.
- Inadequate recruitment.

The coordinating investigator in accordance with the steering committee will decide on exclusion. The investigator and investigating centers have to inform the coordinating investigators immediately if the decision for discontinuation has been made from their side. The reasons for discontinuation should be clarified. Further treatment for the previously included patients should be discussed with the coordinating investigator.

### Strategies to improve adherence to interventions {11c}

To provide the ability for invasively assessing the physiological significance of a coronary artery stenosis using vFFR or FFR, the presence of a catheterization laboratory including all personal and technical requirements is mandatory for the participating centers. In general, vFFR or FFR is performed by specially trained and experienced interventional cardiologists. The required staff is present at all participating centers. Although it is not mandatory, a study nurse may be helpful for the assessment of study endpoints and data handling. The co-investigators should hold appropriate qualification, skills and experience in realizing a clinical trial. Prior to participation, these qualification and skills of all collaborating personal will be revised. In the particular hospitals, as well during investigator meetings, informative functions and special trainings will be organized to guarantee correct execution of the study. Both techniques and the pressure wires are CE-marked and have been developed with the latest techniques. FFR is routinely used in daily clinical practice in patients outlined by the proposed inclusion criteria. Furthermore, the FFR method is recommended in the current guidelines. Clinical follow-up will be evaluated at hospital discharge, as well as at 1 year for the primary study endpoint assessment. In case the patient is not able to return to the PCI center at 1-year follow-up, follow-up can also be performed at the ambulatory cardiologist or general practitioner or by telephone.

### Relevant concomitant care permitted or prohibited during the trial {11d}

The only difference between the treatment arms will be the use of vFFR or FFR. All other clinical decisions will thus be independent of the study. Patients will undergo antiplatelet therapy following current guidelines. Additional medication for the treatment of CAD such as ß-blockers or statins will be administered according to the decision of the individual investigator and common practice of the participating hospitals in accordance with current guidelines [2].

### Provisions for post-trial care {30}

For the general risk of the disease itself and treatment the individual patient is insured by the general hospital insurance. The methods used are standard methods and recommended in current guidelines. Accordingly, no additional study-specific insurance is required.

### Outcomes {12}

The primary endpoint is a composite of major adverse cardiac events (MACE) defined as cardiac death, non-fatal myocardial infarction or any clinically driven unplanned revascularization (PCI or CABG) during the first year after randomization. The diagnosis of myocardial infarction is based on the fourth universal definition of myocardial infarction [18]. Revascularization will be considered “unplanned” when it was not performed as part of the care practice during the index procedure or was not identified at the time of the index procedure as a staged procedure to occur within 60 days.

Repeat revascularization will be performed by PCI or CABG. Furthermore, repeat revascularization will be considered clinically driven if quantitative coronary angiography exhibits stenosis ≥ 50% and one of the following criteria:

- Positive functional study corresponding to the area served by the target lesion,
- Ischemic electrocardiographic changes at rest in a distribution consistent with the target vessel,
- Typical ischemic symptoms corresponding to the target lesions,
- Positive invasive physiologic test (FFR ≤ 0.80 or iFR/RFR ≤ 0.89), or
- Presence of stenosis ≥ 70% diameter, even in the absence of other criteria.

Secondary endpoints include MACE during long-term follow-up at 2 and 5 years, each component of the primary endpoint during 1, 2 and 5 years, any repeat revascularization (PCI or CABG) during 1, 2 and 5 years, all-cause mortality during 1, 2 and 5 years, cross-over rate from one strategy to the other, and the number of analyzable lesions in both treatment arms.

### Participant timeline {13}

**Fig. 3:**
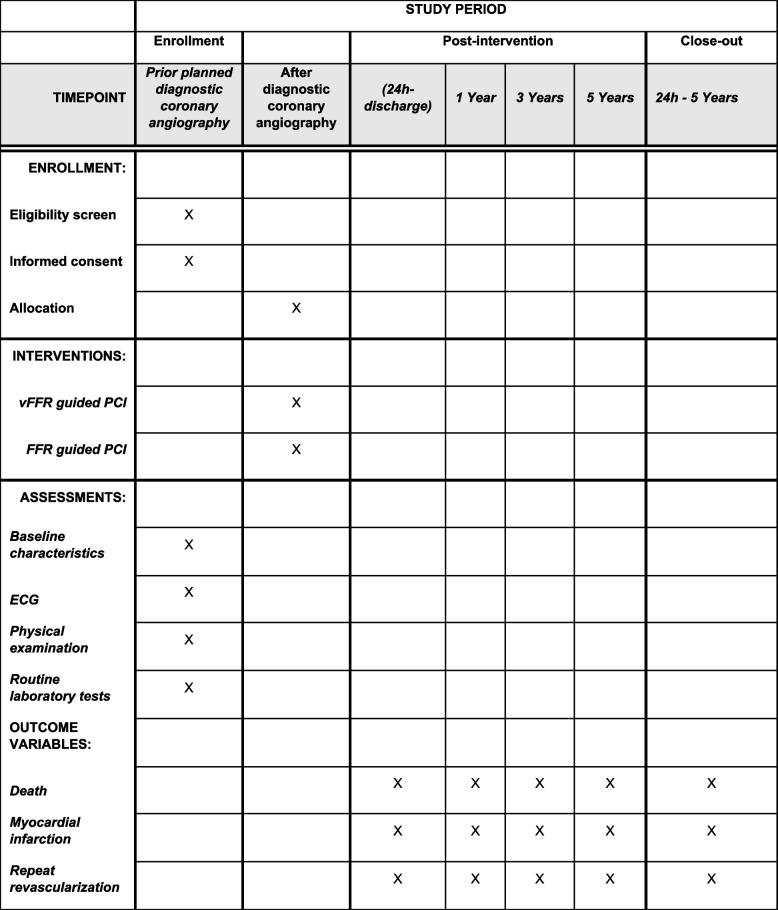
Participant timeline for enrollment, interventions, and assessments. FFR = Fractional Flow Reserve. vFFR = vessel Fractional Flow Reserve

Figure 3 shows the participant timeline.

### Sample size {14}

The prespecified primary hypothesis is that vFFR is non-inferior to FFR to be used as the basis for revascularization decisions for intermediate coronary stenoses regarding the incidence of MACE during a time period of 1 year after randomization.

During the initial planning of the study, we expected a MACE rate of 8% based on the DEFINE-FLAIR study [19]. Given this rate, a sample size of 1888 patients would provide 80% power and a two-sided significance level of 5% to detect non-inferiority of vFFR to FFR, with the use of a non-inferiority margin of 3.5 percentage points for the difference in risk, at a type 1 error rate of 5%. To allow for attrition of 2% at follow-up, the target sample size was initially set at 1926 patients.

Due to slow enrollment and uncertainty of the assumed rate of the primary outcome, the Steering Committee decided to perform a blinded sample-size recalculation on March 2024 [20]. In this analysis, all randomized patients which completed 12 months of follow-up up to December 2023 (*n* = 933) were taken into account. Within this analysis the observed prevalence of the primary endpoint at 12 months after randomization was 4.2%. Taken this MACE rate into account, the recalculation resulted in a reduced sample size of 1032 patients because all other assumptions remained unchanged, in particular the absolute non-inferiority margin of 3.5 percentage points which subsequently results in an increase of the relative non-inferiority margin.

To allow for attrition of approximately 2% at follow-up, the new target sample size was set at 1054 patients.

The blinded sample size recalculation was not prespecified in the protocol but we believe that the statistical interpretability of the study remains fully intact. If the sample size of 1888 patients had remained unchanged, a power of 96% would have been achieved in the end if the event rate of 4.2% had been confirmed. In addition, we assume in the original and updated sample size calculations that the frequency of the primary endpoint is identical in both study arms. This justifies calculating the pooled variance from the observed frequency of the primary endpoint. We also have a large sample size, and the primary endpoint is not that rare. In this scenario, the type I error is only marginally affected.

All sample size calculations were performed using nQuery Adivisor® and SAS 9.4 (double-check) and were based on chi-square test.

### Recruitment {15}

The participating centers are high-volume tertiary care centers with extensive expertise in treatment of patients with CAD and are extensively experienced in performing clinical studies. The estimated inclusion rates of the participating centers are calculated by means of current numbers of patients undergoing PCI and assessment of functional significance of coronary stenoses.

## Assignment of interventions: allocation

### Sequence generation {16a}

Randomization will be performed blockwise with random changing blocks of 4 or 6 using stratification according center in a 1:1 ratio to:

- 1. FFR-guided PCI
  or
- 2. FFR-guided PCI

### Concealment mechanism {16b}

Allocation was concealed by central randomization. The trial was open-label, with the assigned intervention disclosed to both patients and interventionalists immediately after randomization. To minimize bias, data were blinded prior to analysis and centrally analyzed under the supervision of an independent statistician.

### Implementation {16c}

Following informed consent, coronary angiography will be performed. Patients with intermediate stenosis (usually 40–90%) will be randomized by the researchers to either vFFR or FFR-guided therapy. Randomization will be conducted using an established online randomization tool (eBOGEN®) provided by the Institut für Herzinfarktforschung, Ludwigshafen, Germany.

## Assignment of interventions: Blinding

### Who will be blinded {17a}

Blinding of the investigator or patient is not possible due to the type of intervention.

### Procedure for unblinding if needed {17b}

The trial design is open label; therefore, there is no unblinding procedure.

## Data collection and management

### Plans for assessment and collection of outcomes {18a}

To record all pseudonymized findings of each visit, a eCRF will be generated for each patient. The corresponding source documents will be archived in the patients’ record. At the time of screening, baseline and at all other visits, relevant information on history, current medication, vital signs and clinical state will be documented on a separate and specifically prepared sheet.

In case of error detection and subsequent correction, the original entry will remain readable and the correction will be signed and dated by the person authorized to amend. All relevant pages of the eCRF will be controlled for integrity and validity and signed by either the coordinating, principal investigator or a co-investigator.

All relevant study documents including the trial investigator files, the patient identification list, as well as all consent forms and the patient files will be stored for at least 10 years after completion of the last clinical follow-up at the participating centers and the Heart Center Leipzig. Other in-house regulations or legislations demanding longer retention periods (e.g., radiation control regulation, radiation protection law) will be respected.

### Plans to promote participant retention and complete follow-up {18b}

Treatment compliance is not affected by the patient but exclusively by the interventional cardiologist. Based on clinical experience and previous studies, however, it is expected that ~ 97% of all patients will undergo follow-up.

### Data management {19}

Data will be overseen centrally, and all data acquired at the participating centers will be entered in an electronic database at the data management center. Data will be sent to the data management center following randomization as well as after completion of 1, 2 and 5 years follow-up thereafter. The data will be checked for integrity, consistency and plausibility using pre-assigned checks. In case of pending unclarities the affected center will be informed.

Any change on data, for e.g., due to adjustment of pending queries, will be documented through an audit trail of the database.

### Confidentiality {27}

The recorded information includes personal data (e.g., complete name, initials of first and family name, date of birth, address) as well as data on medical treatment and the course of the condition (medical findings, treatment strategies, administered medication, etc.) of the study participants. Patient identifying data will be kept at trial sites only. Trial sites will forward pseudonymous data by eCRF. These data will be electronically stored and analyzed in pseudonymized form (i.e. not directly related to the name of the patient) using a unique identification number. Data processing will be carried out at the Institut für Herzinfarktforschung and also the Leipzig Heart Science (LHS). Protection against unauthorized access and protection against data loss in accordance with data protection acts will be guaranteed and all data are protected from external unauthorized access as only members of the research team are permitted and enabled to access these data. All members of the research team are obliged to secrecy. In case of withdrawal of a prior given data protection waiver all stored data will be deleted. The recorded personal data will be shredded after completion of all study-related projects, at the latest after 10 years in case no legal, statutory or contractual regulations oppose.

### Plans for collection, laboratory evaluation and storage of biological specimens for genetic or molecular analysis in this trial/future use {33}

There are no biological specimens to be collected nor stored.

## Statistical methods

### Statistical methods for primary and secondary outcomes {20a}

All statistical analyses will be conducted by the Institut für Herzinfarktforschung in Ludwigshafen. All analyses will be described in detail in a statistical analysis plan which will be finalized before the randomization of the first patient. The primary analysis is based on all patients with information about primary endpoint (complete 1-year follow-up or cardiac death during 1-year follow-up) using the intention-to-treat (ITT) principle; the per-protocol (PP) principle is used for sensitivity analysis. Adjustment for baseline condition is performed in secondary analyses, as a narrow risk spectrum is included.

The primary analysis will include all patients with information about primary endpoint (complete 1 year follow-up or cardiac death during 1 year follow-up) using the ITT principle. A sensitivity analysis will be performed using the PP principle. The PP population is defined by patients without major protocol violations and treatment received as allocated.

For the calculation of the cumulative incidence function curves, time to death is randomly imputed from the distribution of all observed survival times. Imputation is not provided as standard for secondary endpoints.

Safety will be reported for all included patients by treatment received, rather than allocated.

Non-inferiority of vFFR will be proven by calculating the 97.5% one-sided confidence interval for the difference of event rates in both arms. If the upper confidence bound is below 3.5% (noninferiority margin), non-inferiority is concluded. Secondary endpoints during first year of follow-up will be analyzed by comparing event rates (chi-square or Fisher’s exact test).

Confirmatory statistical test will be performed only for the primary endpoint. The effect on secondary endpoints will be estimated (hazard ratios or odds ratios with corresponding 95% confidence intervals). If *p*-values are calculated for secondary endpoints, they should be interpreted exploratively. Therefore, no adjustments for multiplicity will be performed.

Endpoints during long-term follow-up will be analyzed using time-to-event data (Wilcoxon test). Therefore, lost-to-follow-up patients are taken into consideration. Additionally, Kaplan–Meier curves will be calculated for visualizing primary and secondary endpoints during follow-up. Cox survival analysis will be used to determined independent predictors of the different endpoints.

### Interim analyses {21b}

Initially, neither interim analyses with the objective of premature discontinuation of the trial nor design adaptations are planned. As mentioned above, the Steering Committee decided to perform a sample size re-estimation due to slow recruitment after nearly half of the assumed patients were randomized.

### Methods for additional analyses (e.g., subgroup analyses) {20b}

There are no subgroup analyses planned.

### Methods in analysis to handle protocol non-adherence and any statistical methods to handle missing data {20c}

All patients will remain in their originally allocated arm for the ITT analysis of the primary outcome. A sensitivity analysis will be performed using the PP principle, which will include only patients who received their randomly assigned treatment.

Carefully selected patients based on the inclusion/exclusion criteria will provide a basis for a steady protocol adherence. Treatment compliance is not affected by the patient but exclusively by the interventional cardiologist. Based on clinical experience and previous studies, however, it is expected that ~ 97% of all patients will undergo follow-up.

In general, violation of in- or exclusion criteria is no reason for the individual patient to discontinue participation in the study. If violation of an in- or exclusion criteria at the time of patient recruitment is retrospectively detected, the coordinating investigator will be immediately informed. In agreement with the steering committee, the coordinating investigator will inform the investigational site on how to operate with the individual patient. Documentation of the patient will be continued, unless the patient withdraws his consent.

### Plans to give access to the full protocol, participant-level data and statistical code {31c}

Due to statutory provisions to ensure protection of data quality and verification of study execution, investigators are obligated to provide insight on source data to authorized third parties. This includes monitors, auditors and staff members of the responsible surveillance authorities, all bound to professional secrecy.

## Oversight and monitoring

### Composition of the coordinating center and trial steering committee {5d}

The overall responsibility for the study and its management will be with the coordinating investigator. The steering committee consists of the coordinating investigator, the co-investigators and the principal investigator at each participating site. The coordinating investigator will send approximately every month a newsletter to all members of the steering committee and all study sites summarizing the progress of the study. To ensure the safety interests of the participants, the steering committee will semi-annually evaluate the safety and efficacy of the study intervention, as well as the integrity and validity of the collected data.

### Composition of the data monitoring committee, its role and reporting structure {21a}

Since this is a trial of only moderate size of CE marked devices, no data monitoring committee is defined.

### Adverse event reporting and harms {22}

Adverse event (AE) is any untoward medical occurrence in a patient in the trial and which does not necessarily have to have a causal relationship with the treatment. An AE can therefore be any unfavorable and unintended sign (including an abnormal laboratory finding, for example), symptom, or disease temporally associated with the use of a medicinal product, whether or not considered related to the medicinal product. Only cardiovascular AEs (whether or not the event is considered related to procedure or not) needs reporting. These adverse events will be captured at different time points in the eCRF. A SAE is any untoward medical occurrence that:

- Results in death
- Is life-threatening
- Requires hospitalization or prolongation of existing hospitalization
- Results in persistent or significant disability/incapacity
- Is a congenital anomaly/birth defect

Only cardiovascular SAEs needs reporting. For reporting an electronic form within the eCRF will be provided. The investigator should report all SAEs to the Sponsor as soon as possible after site staff became aware the event met the criteria of an SAE.

The local investigator must assess the seriousness, causality to FFR/vFFR and expectedness of each serious adverse event. After obtaining SAE records from the trial site the sponsor also evaluates seriousness, causality, and expectedness. For this, the investigator might be asked to submit pseudonymized source documents. S(AE)s needs reporting in the time frame from randomization until last follow-up. Pre-existing conditions are not AEs and should not be reported.

### Frequency and plans for auditing trial conduct {23}

For surveillance of the investigating centers on-site monitoring is not planned. In case of high inconclusive data in the eCRF, a risk stratified approach will be performed. In this case at least 5% of data will be randomly selected for validation of the data entered in the eCRF against patients’ source data by the LHS. Monitors will have to ensure that in every individual case informed consent is available before they request access to the patient’s files. Additional monitoring can be carried out if indicated after evaluation of the steering committee in case of insufficient data quality or delayed eCRF input. In the context of these specific monitoring visits, all data relevant for the study will be controlled and eventually updated. To ensure the capability for monitoring, audits, and inspections the investigators will provide access to the study-related facilities and to all files of every study participant to guarantee a complete source data verification. The exact planning and execution of the risk-based monitoring is based on the eligible SOPs of the LHS.

### Plans for communicating important protocol amendments to relevant parties (e.g., trial participants, ethical committees) {25}

After the approval of the central ethics committee, relevant changes of the study protocol can only be conducted if these modifications are again evaluated and accepted by the competent ethics committee(s).

Changes are relevant if they affect:

- The safety of the patients, e.g., essential modifications of therapeutic regimens.
- Additional data assessment or analysis requiring modifications of the informed consent form.
- Interpretation of scientific documents on which the current study is based or which might influence the interpretation of the obtained results.
- The form of administration and conduction of the study.
- The evaluation of the harmlessness of the tested product.

Only the coordinating investigator may carry out changes of the study protocol. However, all coinvestigators can address remarks if modifications seem to be necessary.

### Dissemination plans {31a}

It is aimed to publish the results of this clinical trial in an international peer-reviewed journal. Should the study participants indicate an interest in receiving a summary of the results, they will be shared with them.

## Discussion

Invasive coronary angiography is the reference standard for the diagnosis of CAD. However, visual assessment of epicardial coronary stenoses based solely on angiography is insensitive to assess functional severity and physiologic significance [1].

Most intermediate coronary lesions as visually assessed on angiography are not hemodynamically significant and medical treatment alone is reasonable in these patients [21].

During the last 3 decades, increasing evidence has emerged supporting the use of FFR as the invasive reference method to determine ischemia-producing lesions in epicardial coronary arteries. Therefore, current guidelines on coronary revascularization support the use of FFR to assess the hemodynamic significance of coronary stenoses if there is no prior evidence of ischemia in non-invasive testing [2–4].

Despite clinical benefits of FFR-guided PCI, its application in clinical routine remains limited due to the need to use invasive pressure guidewires requiring administration of adenosine with consecutively prolonged procedure times and complications [5].

The measurement of FFR requires either intravenous or intracoronary administration of adenosine to minimize distal vascular resistance.

The limitation of achieving hyperemia has partly been overcome by the introduction of resting non-hyperemic pressure ratios which eliminate the need for adenosine administration. Non-hyperemic indices such as iFR, dPR, DFR, and RFR show similar discriminatory ability compared to FFR [22]. In this context, the latest European guidelines suggest the use of non-hyperemic iFR as an alternative to standard hyperemic FFR for cardiovascular risk stratification and for the guidance of coronary revascularization [3]. American guidelines include dPR and RFR as further non-hyperemic indices to assess the functional significance of epicardial coronary stenoses [4].

Nevertheless, the use of a pressure wire is still required in all of these techniques. Over the last few years, new angiography-based simulations have emerged for a rapid physiologic assessment of epicardial coronary arteries eliminating the need for pressure wires and hyperemic agents. This was achieved based on (i) three-dimensional reconstruction of the geometry of the target vessel and (ii) applying principles of computational fluid dynamics or simplified mathematical analysis to calculate the pressure drop across the lesions directly using coronary angiography images. The most common methods applied today for the assessment of angiography derived-FFR are quantitative flow ratio (QFR; Medis, The Netherlands), FFR-angio (CathWorks, Kfar-Saba, Israel), FlashAngio caFFR (caFFR; Rainmed Ltd, Suzhou, China), and vessel FFR (vFFR; Pie Medical, Maastricht, The Netherlands). All of these angiography-based indices showed good diagnostic performance in several observational trials as compared to wire-based FFR [7–13]. The existing evidence has recently been summarized in a meta-analysis aiming to compare the diagnostic performance of different angiography-derived FFR systems with wire-based FFR to detect ischemia in patients with intermediate coronary stenoses. A total of 13 studies (1842 vessels) including different approaches for calculation of angiography-derived FFR were included. Taken together, angiography-derived FFR showed high sensitivity (89%) and specificity (90%) to detect hemodynamically relevant coronary stenoses. The summary area under the receiver operating curve (AUC) was 0.84. All three angiography-based FFR approaches analyzed showed good diagnostic performance without any differences between the methods for FFR calculation [6].

Specifically, the diagnostic accuracy of vFFR applied in the LIPSIA-STRATEGY trial as well its correlation with classically measured FFR has been confirmed by several observational studies. The first validation of this technique was published in the FAST I (Fast Assessment of STenosis severity) study, an observational, retrospective, single-center trial including 100 patients with stable angina or NSTE-ACS. A good linear correlation was found between vFFR and wire guided-FFR (*r* = 0.89; *p* < 0.001) with a notably low inter-observer variability [7]. Thereafter, FAST EXTEND retrospectively assessed 296 patients. This study confirmed the good diagnostic performance of vFFR and close correlation between vFFR and FFR, which appeared to be consistent among different vessel and anatomy subsets [11].

Main limitations of these studies were the retrospective nature and the off-line vFFR assessment. The first prospective study, FAST II enrolled 334 patients and compared the performance of vFFR with FFR. vFFR was measured both offline by a core-lab and online in the catheterization laboratory. vFFR showed good diagnostic performance and accuracy in identifying the functional relevance of coronary lesions compared with invasive wire-based FFR (AUC 0.93; 95% CI 0.90–0.96; *p* < 0.001). In addition, both vFFR measurements (offline and online) showed good correlation with FFR as reference (offline *r* = 0.74, *p* < 0.001 and online *r* = 0.76, *p* < 0.001) [12].

QFR is another angiography-based method which was compared to wire-based FFR. In the multicenter FAVOR Pilot Study, QFR and FFR of 84 vessels in 73 patients with intermediate coronary lesions were compared. This study showed that a QFR model which does not require induction of hyperemia correlates well with FFR. The overall diagnostic accuracy for identifying an FFR of ≤ 0.80 was more than 80% [8]. Similarly, the FAVOR II China Study which assessed QFR and FFR in a total of 328 vessels of 308 patients showed on both patient- and vessel-level that QFR analysis had nearly 93% diagnostic accuracy using FFR as a reference [9]. Another recent multicenter registry showed excellent diagnostic performance and agreement of QFR compared to FFR (*r* = 0.860, *p* < 0.001). In addition, the prognostic implications of QFR in terms of a composite of cardiac death, target-vessel myocardial infarction, and ischemia-driven target lesion revascularization at 2 years were tested. A QFR value ≤ 0.80 had a significantly higher risk for the occurrence of the composite endpoint than vessels with a QFR value > 0.80 (4.2% vs. 0.9%, *p* = 0.022) [10].

In the multicenter randomized FAVOR III China trial, a total of 3825 patients with predominantly unstable angina or chronic stable angina and at least one intermediate coronary stenosis (diameter stenosis of 50–90%) were randomized to a QFR-guided strategy (PCI in lesions with an QFR ≤ 0.80) or an angiography-guided strategy (PCI based on standard visual assessment). QFR-guided revascularization was associated with an improved 1- and 2-year clinical outcome in respect of the occurrence of MACE (a composite of death from any cause, myocardial infarction, or ischemia-driven revascularization) compared with those undergoing angiography-guided revascularization [23, 24]. A recent network meta-analysis of fifteen randomized trials including 16,333 patients with chronic stable angina or acute coronary syndrome and coronary stenosis > 30% eligible for PCI compared different strategies (angiography, FFR, iFR, intravascular imaging, and QFR) to guide decision making to perform PCI. The analysis demonstrated that QFR-based revascularization was associated with a decreased rate of MACE when compared to angiography-guided revascularization. Interestingly, QFR-based revascularization was also superior compared to a wire-based (iFR and FFR) revascularization strategy [25]. These hypothesis-generating observations strengthen the need for prospective randomized trials comparing an image-based FFR technique with the current gold standard FFR.

An ongoing prospective, randomized trial (FAVOR III Europe Japan; NCT03729739) will test whether a QFR-based approach is non-inferior (in terms of clinical outcome data) to a standard FFR guided approach in the assessment of intermediate coronary stenoses. Table 1 shows an overview of ongoing trials comparing different angiography-based FFR indices with invasive FFR.

**Table 1:** Ongoing randomized trials. POCE = patient-oriented composite endpoint (all-cause death, any stroke, any myocardial infarction, any clinically and physiology driven revascularization). MACE = major adverse cardiac events (all-cause death, any myocardial infarction, any revascularization). DAPT = dual-antiplatelet therapy

**Study name**	**FAST III**	**LIPSIA-STRATEGY**	**FAVOR III Eu-Japan**	**PIONEER IV**	**Flash FFR II**	**All-Rise**
**Device**	vFFR	vFFR	QFR	QFR	CaFFR	FFRangio
**Comparator**	FFR	FFR	FFR	Usual Care	FFR	FFR
**Trial design**	Non-inferiority	Non-inferiority	Non-inferiority	Non-inferiority	Non-inferiority	Non-inferiority
**Region**	Europe	Germany	Europe	Europe	China	USA Israel
**Nr of patients**	2228	1054	2000	2540	2132	1924
**Location/sites**	Europe/35	Germany/8	Europe/39 Japan/2	Europe/30	China/13	USA/17 Israel/1
**Power:** **α:** **Non-inf. Margin:** **Event rate inv. arm:** **Event rate control arm:**	80% 0.025 3.0% 6.5% 6.5%	80% 0.025 3.5% 4.2% 4.2%		90% 0.05 3.2% 8% 8%		
**Inclusion criteria (lesion)**	Lesion diameter ≥30 - ≤80%	Lesion diameter ≥40 - ≤90%	Lesion diameter ≥40 - ≤90% Ref diam ≥ 2.5	Lesion diameter ≥50% Ref diam ≥ 2.25	Lesion diameter ≥50 - ≤90% Ref diam ≥ 2.25	Lesion diameter ≥50 - ≤90%
**Primary Outcome**	MACE within 12 months	MACE within 12 months	MACE within 12 months	POCE within 12 months	MACE within 12 months	MACE within 12 months
**Specifics**				Unrestricted use of the HT Supreme sirolimus-eluting stent (SINOMED, Tianjin, China) DAPT (aspirin + ticagrelor) for 1 month, followed by 11 months of ticagrelor only		
**Clinical Trial ID**	NCT04931771	NCT03497637	NCT03729739	NCT04923191	NCT04575207	NCT05893498

As mentioned above the Steering Committee decided to perform a sample size re-estimation due to slow recruitment after nearly half of the assumed patients were randomized. Taken the observed event rate into account re-calculation resulted in a decrease of the total sample size.

However, the limitation is that—with an unchanged absolute non-inferiority margin of 3.5 percentage points—an increased (1.44 to 1.83) relative non-inferiority margin must be accepted. Although blinded recalculation was initially not part of the study protocol this method enables a methodologically sound premature finalization of the study.

However, and importantly, to date no randomized clinical trial has compared an image-based FFR methodology with standard invasive FFR in terms of clinical outcomes.

The randomized multicenter LIPSIA-STRATEGY trial will be the first to compare angiography-derived vFFR with invasive FFR with respect to clinical outcomes in patients with intermediate coronary lesions.

## Trial status

Recruitment started in June 2020. Based on the updated sample size, enrolment was completed in February 2025.

## Data Availability

Any data used during the current study will be made available upon reasonable request if all members of the investigative team approve the request.

## Abbreviations

CAD: Coronary artery disease
ACS: Acute coronary syndrome
AUC: Area under the receiver operating curve
FFR: Fractional flow reserve
PCI: Percutaneous coronary intervention
CABG: Coronary artery bypass graft
iFR: Instantaneous wave-free ratio
dPR: Diastolic pressure ratio
RFR: Resting full-cycle ratio
DFR: Diastolic hyperemia-free ratio
QFR: Quantitative flow ratio
CAAS vFFR: Cardiovascular Angiographic Analysis Systems for vessel Fractional Flow Reserve
SAE: Serious adverse event
AE: Adverse event
eCRF: Electronic Case Report Forms
QCA: Quantitative coronary angiography
MACE: Major adverse cardiac event
POCE: Patient-oriented composite endpoint
LHS: Leipzig Heart Science
ITT: Intention to treat
PP: Per protocol

## References

[1] Park SJ, Kang SJ, Ahn JM, Shim EB, Kim YT, Yun SC, et al. Visual-functional mismatch between coronary angiography and fractional flow reserve. J Am Coll Cardiol Intv. 2012;5:1029–36. 10.1016/j.jcin.2012.07.007 23078732

[2] Neumann FJ, Sousa-Uva M, Ahlsson A, Alfonso F, Banning AP, Benedetto U, et al. 2018 ESC/EACTS guidelines on myocardial revascularization. Eur Heart J. 2019;40:87–165. 30165437 10.1093/eurheartj/ehy394

[3] Knuuti J, Wijns W, Saraste A, Capodanno D, Barbato E, Funck-Brentano C, et al. Esc guidelines for the diagnosis and management of chronic coronary syndromes. Eur Heart J. 2020;41:407–77. 31504439 10.1093/eurheartj/ehz425

[4] Patel MR, Calhoon JH, Dehmer GJ, Grantham JA, Maddox TM, Maron DJ, et al. ACC/AATS/AHA/ASE/ASNC/SCAI/SCCT/STS 2017 appropriate use criteria for coronary revascularization in patients with stable ischemic heart disease: a report of the American College of Cardiology Appropriate Use Criteria Task Force, American Association for Thoracic Surgery, American Heart Association, American Society of Echocardiography, American Society of Nuclear Cardiology, Society for Cardiovascular Angiography and Interventions, Society of Cardiovascular Computed Tomography, and Society of Thoracic Surgeons. J Thorac Cardiovasc Surg. 2019;157:e131–61. 33198024 10.1016/j.jtcvs.2018.11.027

[5] Dattilo PB, Prasad A, Honeycutt E, Wang TY, Messenger JC. Contemporary patterns of fractional flow reserve and intravascular ultrasound use among patients undergoing percutaneous coronary intervention in the United States: insights from the National Cardiovascular Data Registry. J Am Coll Cardiol. 2012;60:2337–9. 23194945 10.1016/j.jacc.2012.08.990

[6] Collet C, Onuma Y, Sonck J, Asano T, Vandeloo B, Kornowski R, et al. Diagnostic performance of angiography-derived fractional flow reserve: a systematic review and Bayesian meta-analysis. Eur Heart J. 2018;39:3314–21. 30137305 10.1093/eurheartj/ehy445

[7] Masdjedi K, van Zandvoort LJC, Balbi MM, Gijsen FJH, Ligthart JMR, Rutten MCM, et al. Validation of a three-dimensional quantitative coronary angiography-based software to calculate fractional flow reserve: the FAST study. EuroIntervention. 2020;16:591–9. 31085504 10.4244/EIJ-D-19-00466

[8] Tu S, Westra J, Yang J, von Birgelen C, Ferrara A, Pellicano M, et al. Diagnostic accuracy of fast computational approaches to derive fractional flow reserve from diagnostic coronary angiography: the International Multicenter FAVOR Pilot Study. JACC Cardiovasc Interv. 2016;9:2024–35. 27712739 10.1016/j.jcin.2016.07.013

[9] Xu B, Tu S, Qiao S, Qu X, Chen Y, Yang J, et al. Diagnostic accuracy of angiography-based quantitative flow ratio measurements for online assessment of coronary stenosis. J Am Coll Cardiol. 2017;70:3077–87. 29101020 10.1016/j.jacc.2017.10.035

[10] Choi KH, Lee SH, Lee JM, Hwang D, Zhang J, Kim J, et al. Clinical relevance and prognostic implications of contrast quantitative flow ratio in patients with coronary artery disease. Int J Cardiol. 2021;325:23–9. 32910999 10.1016/j.ijcard.2020.09.002

[11] Neleman T, Masdjedi K, Van Zandvoort LJC, Tomaniak M, Ligthart JMR, Witberg KT, et al. Extended validation of novel 3D quantitative coronary angiography-based software to calculate vFFR: the FAST EXTEND study. JACC Cardiovasc Imaging. 2021;14:504–6. 33011122 10.1016/j.jcmg.2020.08.006

[12] Masdjedi K, Tanaka N, Van Belle E, Porouchani S, Linke A, Woitek FJ, et al. Vessel fractional flow reserve (vFFR) for the assessment of stenosis severity: the FAST II study. EuroIntervention. 2022;17:1498–505. 34647890 10.4244/EIJ-D-21-00471 PMC9896401

[13] Li J, Gong Y, Wang W, Yang Q, Liu B, Lu Y, et al. Accuracy of computational pressure-fluid dynamics applied to coronary angiography to derive fractional flow reserve: FLASH FFR. Cardiovasc Res. 2020;116:1349–56. 31693092 10.1093/cvr/cvz289

[14] Pijls NH, De Bruyne B, Peels K, Van Der Voort PH, Bonnier HJ, Bartunek J, et al. Measurement of fractional flow reserve to assess the functional severity of coronary-artery stenoses. N Engl J Med. 1996;334:1703–8. 8637515 10.1056/NEJM199606273342604

[15] Morris PD, Narracott A, von Tengg-Kobligk H, Silva Soto DA, Hsiao S, Lungu A, et al. Computational fluid dynamics modelling in cardiovascular medicine. Heart. 2016;102:18–28. 26512019 10.1136/heartjnl-2015-308044 PMC4717410

[16] Morris PD, Ryan D, Morton AC, Lycett R, Lawford PV, Hose DR, et al. Virtual fractional flow reserve from coronary angiography: modeling the significance of coronary lesions: results from the VIRTU-1 (VIRTUal Fractional Flow Reserve From Coronary Angiography) study. J Am Coll Cardiol. 2013;6:149–57. 10.1016/j.jcin.2012.08.024 23428006

[17] Morris PD, Silva Soto DA, Feher JFA, Rafiroiu D, Lungu A, Varma S, et al. Fast virtual fractional flow reserve based upon steady-state computational fluid dynamics analysis: results from the VIRTU-Fast study. JACC Basic Transl Sci. 2017;2:434–46. 28920099 10.1016/j.jacbts.2017.04.003 PMC5582193

[18] Thygesen K, Alpert JS, Jaffe AS, Executive Group on behalf of the Joint European Society of Cardiology (ESC)/American College of Cardiology (ACC)/American Heart Association (AHA)/World Heart Federation (WHF) Task Force for the Universal Definition of Myocardial Infarction, et al. Fourth universal definition of myocardial infarction (2018). Circulation. 2018;138:618–51.

[19] DEFINE-FLAIR Trial Investigators, Lee JM, Choi KH, Koo BK, Dehbi HM, Doh JH, et al. Use of the instantaneous wave-free ratio or fractional flow reserve in PCI. N Engl J Med. 2017;376:1824–34. 28317458 10.1056/NEJMoa1700445

[20] Friede T, Kieser M. Blinded sample size reassessment in non-inferiority and equivalence trials. Stat Med. 2003;22:995–1007. 12627414 10.1002/sim.1456

[21] Tonino PA, Fearon WF, De Bruyne B, Oldroyd KG, Leesar MA, Ver Lee PN, et al. Angiographic versus functional severity of coronary artery stenoses in the fame study: fractional flow reserve versus angiography in multivessel evaluation. J Am Coll Cardiol. 2010;55:2816–21. 20579537 10.1016/j.jacc.2009.11.096

[22] Lee JM, Choi KH, Park J, Hwang D, Rhee TM, Kim J, et al. Physiological and clinical assessment of resting physiological indexes. Circulation. 2019;139:889–900. 30586749 10.1161/CIRCULATIONAHA.118.037021

[23] Xu B, Tu S, Song L, Jin Z, Yu B, Fu G, et al. Angiographic quantitative flow ratio-guided coronary intervention (FAVOR III China): a multicentre, randomised, sham-controlled trial. Lancet. 2021;398:2149–59. 34742368 10.1016/S0140-6736(21)02248-0

[24] Song L, Xu B, Tu S, Guan C, Jin Z, Yu B, et al. 2-year outcomes of angiographic quantitative flow ratio-guided coronary interventions. J Am Coll Cardiol. 2022;80:2089–101. 36424680 10.1016/j.jacc.2022.09.007

[25] d’Entremont MA, Tiong D, Sadeghirad B, McGrath BP, Cioffi GM, Garni TA, et al. Assessment of coronary stenoses for percutaneous coronary intervention: a systematic review and network meta-analysis of randomized trials. Am J Cardiol. 2024;223:29–39. 38768846 10.1016/j.amjcard.2024.05.019

